# A non-solvated form of [(*Z*)-*O*-methyl-*N*-(2-methyl­phen­yl)­thio­carbamato-κ*S*](tri­phenyl­phosphane-κ*P*)gold(I): crystal structure and Hirshfeld surface analysis

**DOI:** 10.1107/S2056989016014419

**Published:** 2016-09-16

**Authors:** Chien Ing Yeo, Sang Loon Tan, Edward R. T. Tiekink

**Affiliations:** a Research Centre for Crystalline Materials, Faculty of Science and Technology, Sunway University, 47500 Bandar Sunway, Selangor Darul Ehsan, Malaysia

**Keywords:** crystal structure, gold, thio­carbamate, solvatomorphs, Hirshfeld surface analysis

## Abstract

The title compound, [Au(C_9_H_10_NOS)(C_18_H_15_P)], features a near linear P—Au—S arrangement defined by phosphane P and thiol­ate S atoms with the minor distortion from the ideal [P—Au—S is 177.61 (2)°] being traced in part to the close intra­molecular approach of an O atom [Au⋯O = 3.040 (2) Å]. The packing features supra­molecular layers lying parallel to (011) sustained by a combination of C—H⋯π and π–π [inter-centroid distance = 3.8033 (17) Å] inter­actions. The mol­ecular structure and packing are compared with those determined for a previously reported hemi-methanol solvate [Kuan *et al.* (2008 ▸). *CrystEngComm*, **10**, 548–564]. Relatively minor differences are noted in the conformations of the rings in the Au-containing mol­ecules. A Hirshfeld surface analysis confirms the similarity in the packing with the most notable differences relating to the formation of C—H⋯S contacts between the constituents of the solvate.

## Abstract 2

A near linear geometry for the gold(I) atom defined by a P, S donor set is found in the title compound; an intra­molecular Au⋯O short contact is noted. Supra­molecular layers sustained by C—H⋯π and π—π inter­actions feature in the crystal.

## Chemical context   

Triorganophosphanegold(I) carbonimido­thio­ates, *i.e*. mol­ecules of the general formula *R*_3_PAu[SC(O*R*′)=N*R*′′ for *R*, *R*′ and *R*′′ = alkyl, aryl, were first described in 1993 as were the crystal and mol­ecular structures of archetypal Ph_3_PAu[SC(OMe)=NPh (Hall *et al.*, 1993 ▸). Since then, approximately 70 crystal structures, including those of bident­ate phosphanes and bipodal analogues, have been described in the crystallographic literature (Groom *et al.*, 2016 ▸). The inter­est in phosphanegold(I) carbonimido­thio­ates stems from two distinct considerations related to their relatively facile synthesis, their long-term stability and their readiness to crystallize, namely crystal engineering and evaluation for biological activity. In the former and reflecting their propensity to form diffraction-quality crystals, an unprecedented comprehensive series of compounds, *R*_3_PAu[SC(OMe)=NC_6_H_4_NO_2_-*p*] (*R* = Et, Cy and Ph), and bidentate phosphane analogues, Ph_2_P–(CH_2_)_*n*_–PPh_2_ for *n* = 1–4 and for when the bridge is ferrocenyl, enabled correlations between the formation of Au⋯Au (aurophilic) inter­actions and solid-state luminescence responses (Ho *et al.*, 2006 ▸). In another series of compounds where the diphosphane ligand was held constant, *i.e*. [(Ph_2_P(CH_2_)_4_PPh_2_){AuSC(O*R*′)=NC_6_H_4_ *Y*-*p*}_2_] for *R*′ = Me, Et or *i*Pr and *Y* = H, NO_2_ or Me, the packing was assessed in terms of delineating the influence of *R*′ and *Y* substituents (Ho & Tiekink, 2007 ▸). In yet another systematic series of compounds, *i.e*. of the general formula *R*_3_PAu[SC(OMe)=N*R*′′], for *R* = Ph, *o*-tol, *m*-tol or *p*-tol, and *R*′′ = Ph, *o*-tol, *m*-tol, *p*-tol or C_6_H_4_NO_2_-*p*, it was possible to assess the impact of steric and electronic effects upon the formation of intra­molecular Au⋯O or Au⋯π(N-bound ring) inter­actions (Kuan *et al.*, 2008 ▸). Over and above these studies, phosphanegold(I) carbonimido­thio­ates exhibit promising biological potential in the context of anti-cancer activity (Yeo, Ooi *et al.*, 2013 ▸; Ooi *et al.*, 2015 ▸) and anti-microbial activity (Yeo, Sim *et al.*, 2013 ▸). Just as systematic variations in the substituents influences the mol­ecular packing, this also influences biological effects so that, for example, different apoptotic mechanisms of cell death are induced when the O-bound *R*′ is varied. It was in fact during biological investigations that the title compound, Ph_3_PAu[SC(OMe)=N(*o*-tol)] (I), was prepared once again, having been previously characterized as a 1:1 hemi-methanol solvate (I·0.5MeOH; Kuan *et al.*, 2008 ▸). Herein, the crystal and mol­ecular structures of (I) are described along with Hirshfeld surface analyses of both (I) and (I·0.5MeOH).
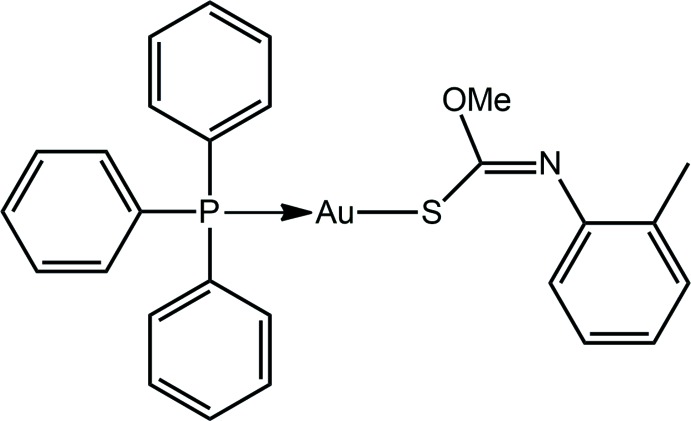


## Structural commentary   

**Figure 1:**
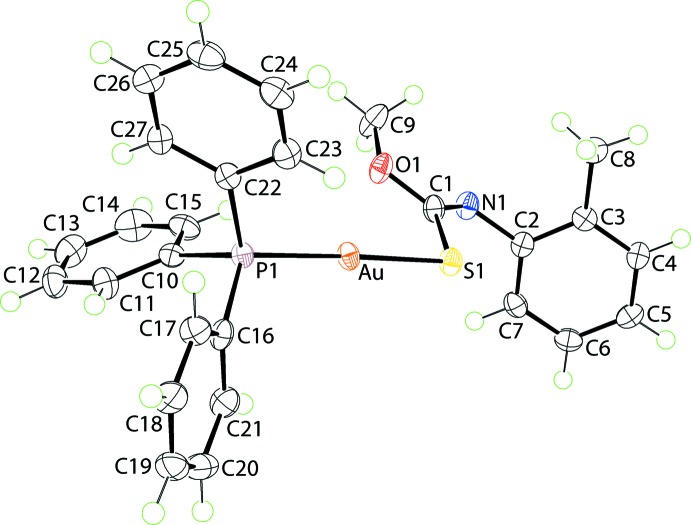
Mol­ecular structure of (I), showing the atom-labelling scheme and displacement ellipsoids at the 70% probability level.

The gold(I) atom in (I), Fig. 1 ▸, exists within the anti­cipated linear geometry defined by thiol­ate-S1 and phosphane-P1 atoms. Support for the ‘thiol­ate-S1’ assignment comes about by the elongation of the C1—S1 bond to 1.768 (3) Å, Table 1 ▸, *c.f.* 1.6700 (14) Å, and contraction of the C1—N1 bond in (I) to 1.260 (3) Å, *c.f.* 1.3350 (15) Å in the structure of the non-coordinating mol­ecule, *i.e*. S=C(OMe)N(H)(*o*-tol) (Kuan *et al.*, 2005 ▸). The small deviation from linearity about the gold(I) atom [P—Au—S = 177.61 (2)°] may be related to the close approach of the O1 atom, Au⋯O1 is 3.040 (2)°, as the carbonimido­thio­ate ligand is orientated to place the oxygen atom in close proximity to the gold atom, Fig. 1 ▸. There are also significant differences in key angles between the coordinating and non-coordinating forms of the ligand, especially about the C1 atom. These reflect the reorganization of π-electron density manifested in the C=N and C=S bonds, respectively. Thus, the widest angles in the anion involve C=N and those in the free mol­ecule, involve C=S. A relatively large change is noted for the C1—N1—C2 angles, *i.e*. 121.4 (2) and 127.11 (12)°, respectively, for the coordinating and non-coord­inating ligands, which is a result of the presence of the acidic proton in the latter. In terms of conformation of the anion in (I), the central residue comprising the S1, O1, N1 and C1 atom is strictly planar (r.m.s. deviation of the fitted atoms = 0.0091 Å), with the pendent C2 and C9 atoms lying 0.035 (4) and 0.198 (4) Å out of this plane, respectively. The dihedral angle between the central residue and the N-bound aryl ring is 85.08 (7)°, indicating a nearly perpendicular arrangement; in the free ligand the comparable angle is 51.84 (6)° (Kuan *et al.*, 2005 ▸).

Salient geometric parameters for (I·0.5MeOH) (Kuan *et al.*, 2008 ▸) are also included in Table 1 ▸. From these data, it is apparent there are no great variations between the structures with perhaps the exception of the Au—S1 bond length in (I) being 0.01 Å longer than in (I·0.5MeOH). In terms of angles, the angle subtended at the S1 atom is about 2° tighter in (I). The intra­molecular Au⋯O1 separation is 0.05 Å shorter in (I) but the deviation from linearity is less, reflecting the weak nature of this inter­action.

**Figure 2:**
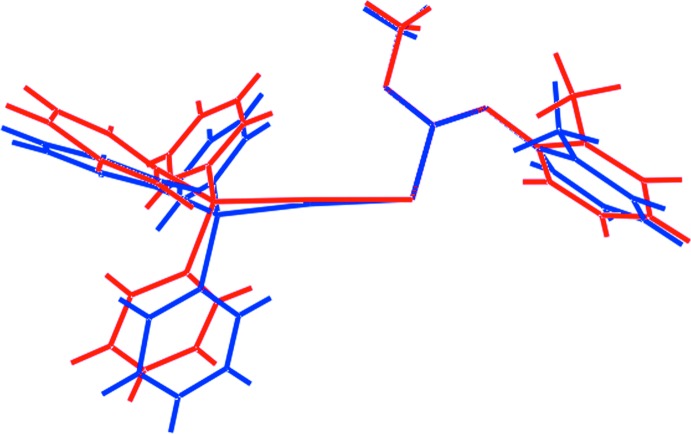
Overlay diagram of (I) (red image) and I in (I·0.5MeOH) (blue). The mol­ecules have been overlapped so that the S1, O1 and N1 atoms are coincident.

Fig. 2 ▸ shows an overlay diagram for (I) and I in (I·0.5MeOH). From this it can be seen there is evidently a close overlap of all but the aryl rings that display orientational differences.

## Supra­molecular features   

**Figure 3:**
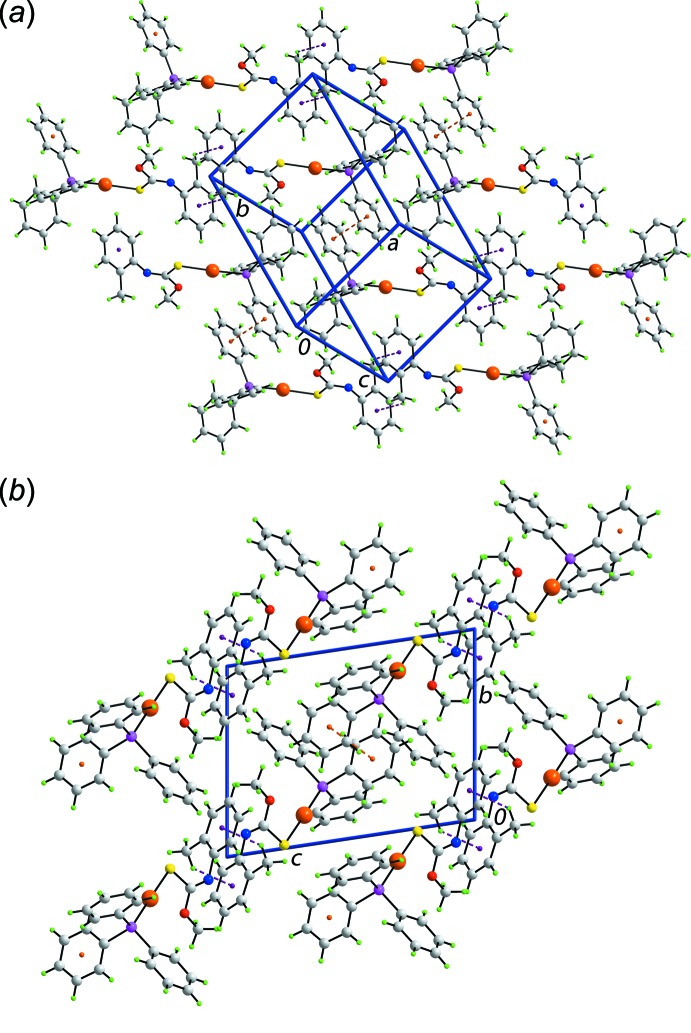
Mol­ecular packing in (I): (*a*) a view of the supra­molecular layer sustained by C—H⋯π and π–π contacts, shown as purple and orange dashed lines, respectively, and (*b*) a view of the unit-cell contents shown in projection down the *a* axis, highlighting the stacking of (011) layers.

In the crystal of (I), the most prominent points of contact between mol­ecules are of the type C—H⋯π and π–π, Table 2 ▸. Thus, centrosymmetrically related *o*-tolyl residues associate *via* pairs of methyl-C—H⋯π(*o*-tol) inter­actions, and centrosymmetrically related phosphane ligands are connected *via* face-to-face π–π inter­actions involving one of the P-bound phenyl rings only. The result is the formation of supra­molecular layers lying parallel to (011) as illustrated in Fig. 3 ▸ *a*. The layers stack with no directional inter­actions between them, Fig. 3 ▸ *b*.

**Figure 4:**
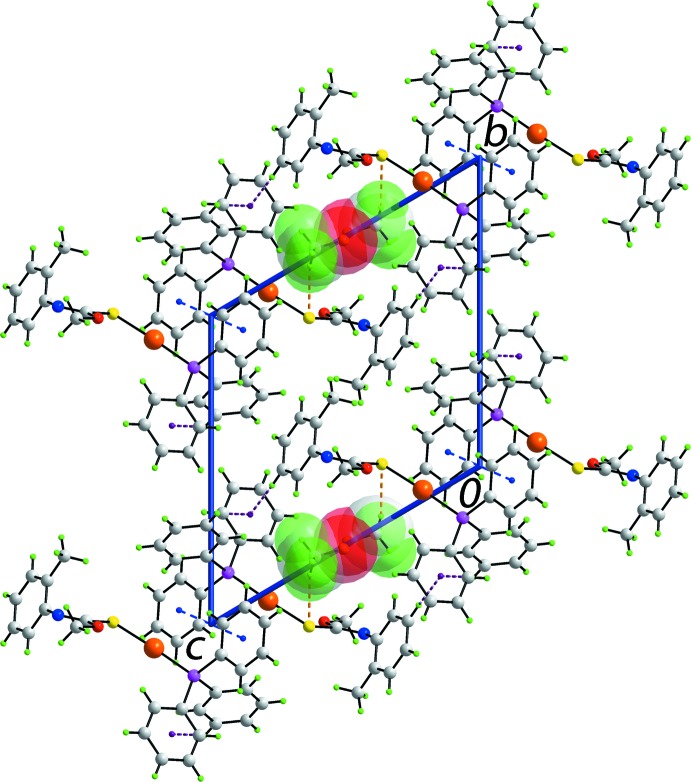
Mol­ecular packing in (I·0.5MeOH): a view of the unit-cell contents shown in projection down the *a* axis. The C—H⋯S, C—H⋯π and π–π contacts are shown as orange, purple and blue dashed lines, respectively. The methanol mol­ecules are highlighted in space-filling mode.

The packing of (I·0.5MeOH) is also characterized by supra­molecular layers. These are sustained by π–π inter­actions of 3.687 (4) Å between centrosymmetrically related mol­ecules in a face-to-face fashion, as for (I), and by phenyl- and *o*-tolyl-C—H⋯π(P-phen­yl) inter­actions. The layers stack along the *b* axis devoid of specific inter­actions between successive layers. This arrangement defines columns along the *a* axis in which reside the disordered methanol mol­ecules, Fig. 4 ▸. The partially occupied methanol mol­ecules in (I·0.5MeOH), disordered over a centre of inversion, are connected to the host framework *via* methyl-C—H⋯S inter­actions.

## Analysis of the Hirshfeld surfaces   

Hirshfeld surface analysis and fingerprint plots were undertaken to study the inter­molecular contacts and topological differences between (I) and its methanol hemi-solvate, (I·0.5MeOH). Briefly, the inter­nal (*d*_i_) and external (*d*_e_) distances of atomic surface points to the nearest nucleus were computed for the mol­ecules in both (I) and (I·0.5MeOH) (Spackman & Jayatilaka, 2009 ▸; McKinnon *et al.*, 2007 ▸). The resulting normalized contact distances (*d*_norm_) were mapped on the Hirshfeld surface in the range −1.04 to 1.91 Å. The contact distances shorter than the sum of van der Waals radii are highlighted in red while distances equal to or longer than the sum of van der Waals radii are shown in white and blue, respectively (McKinnon *et al.*, 2007 ▸). The combination of *d*_i_ and *d*_e_ in inter­vals of 0.01 Å result in the two-dimensional fingerprint plots, where the different colours on the fingerprint plots represent the probability of occurrence, ranging from blue (few points) through green to red (many points) (Spackman & McKinnon, 2002 ▸). All analyses were performed using *Crystal Explorer* (Wolff *et al.*, 2012 ▸).

The number of Hirshfeld surfaces that are unique in a given crystal structure depends on the number of independent mol­ecules in the asymmetric unit (Fabbiani *et al.*, 2007 ▸). For this reason, the Hirshfeld surfaces for (I·0.5MeOH) were modelled separately for (I) and for MeOH, while the Hirshfeld surface of (I·0.5MeOH), as a whole, were also included for a thorough comparison of the mol­ecular packing in (I) and (I·0.5MeOH).

**Figure 5:**
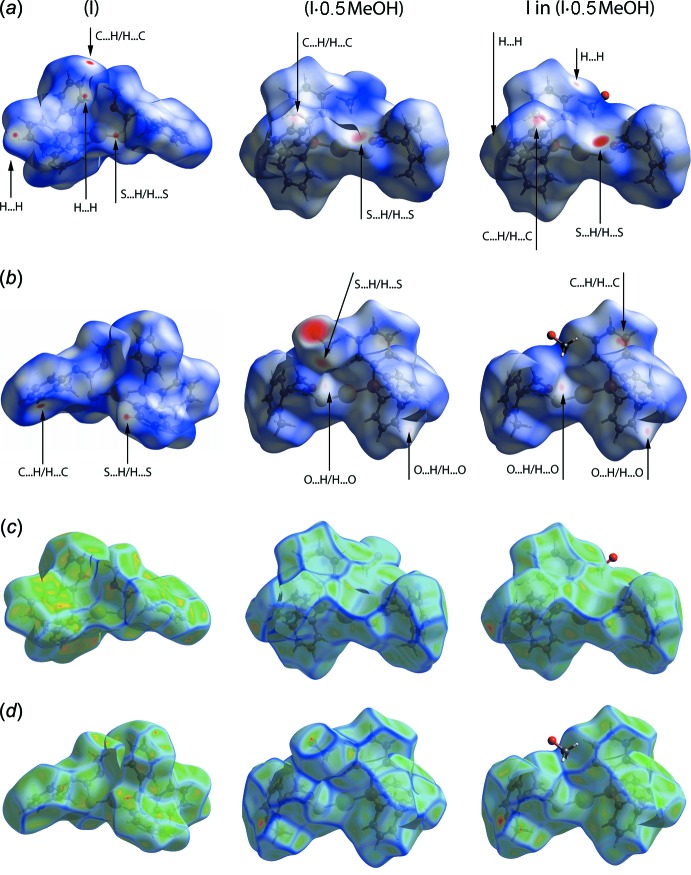
Comparison between (I), (I·0.5MeOH) and I in (I·0.5MeOH) of (*a*) the front view of the complete Hirshfeld surface, (*b*) the back view of the complete Hirshfeld surface, (*c*) the front view of the curvedness and (*d*) the back view of the curvedness.

Fig. 5 ▸ *a* and 5*b* show the front and back views of Hirshfeld surfaces for (I), (I·0.5MeOH) as well as for I in (I·0.5MeOH) which are displayed in approximately the same orientation. Despite the presence of additional solvent mol­ecule in (I·0.5MeOH), both this and (I) are governed by similar inter­molecular contacts as can be observed through the appearance of several red spots on the Hirshfeld surfaces of both structures. These are mainly attributed to H⋯H, C⋯H/H⋯C and S⋯H/H⋯S contacts. However, a close inspection of the Hirshfeld surface of I in (I·0.5MeOH) reveals a stark difference as compared to (I), in that evidence is found for a close contact through a S⋯H inter­action with the solvent MeOH mol­ecule as readily seen from the intense red spot in Fig. 5 ▸ *a* – right. Apart from this contact, I in (I·0.5MeOH) also forms weak inter­action, as demonstrated by the less intense red spot in Fig. 5 ▸ *b* – right, through O⋯H with another mol­ecule of I but beyond the sum of their van der Waals radii (Spek, 2009 ▸).

In view that the conformational flexibility highlighted in Fig. 2 ▸, the mapping of curvedness over the Hirshfeld surface was undertaken in order to correlate these with some physico­chemical properties. Fig. 5 ▸ *c* and 5*d* show the front and back views of the curvedness for (I), (I·0.5MeOH) and I in (I·0.5MeOH). From these views, it is clear (I) exhibits a relatively broad region of curvedness surface, Fig. 5 ▸ *c* – left. It is presumably for this reason that (I) has a relatively greater surface area, indicating a more compact conformation, *i.e*. having a lower volume, and is more densely packed than I in (I·0.5MeOH), see data in Table 3 ▸. Inter­estingly, it seems the mol­ecular shape exerts a great influence over the inter­molecular inter­actions and the density of the resultant crystal structures, Table 3 ▸. The packing efficiency of (I) is also greater than that of (I·0.5MeOH), suggesting that the incorporation of methanol in the mol­ecular packing of (I·0.5MeOH) is not directed by the need to fill otherwise free space in (I).

**Figure 6:**
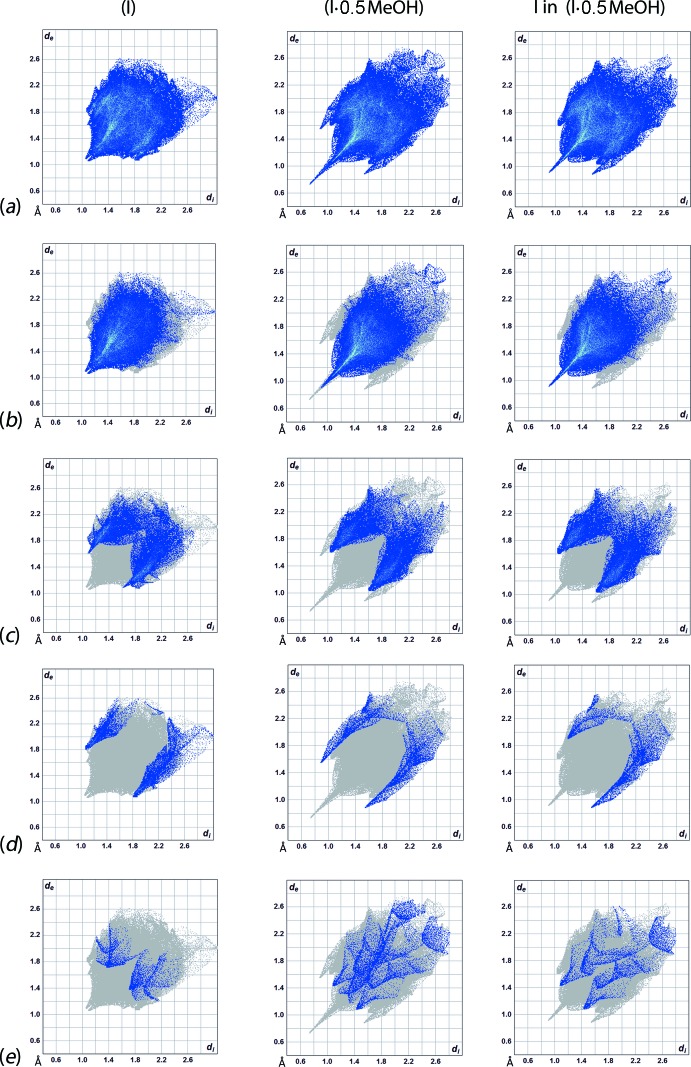
Comparison between (I), (I·0.5MeOH) and I in (I·0.5MeOH) of (*a*) the full fingerprint plots, and delineated two-dimensional plots associated with (*b*) H⋯H, (*c*) C⋯H/H⋯C, (*d*) S⋯H/H⋯S and (*e*) O⋯H/H⋯O contacts.

**Figure 7:**
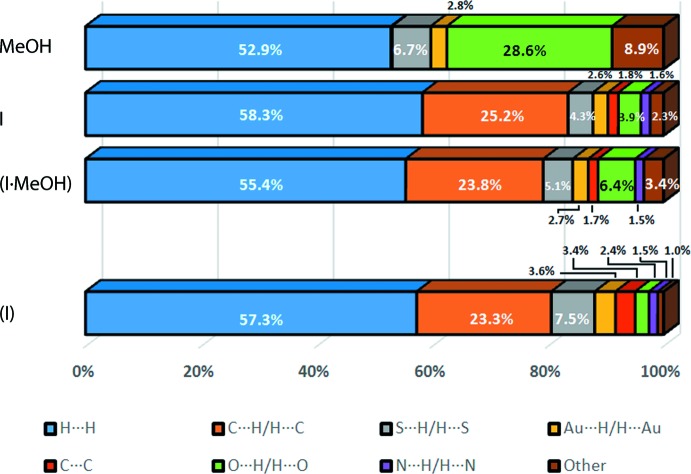
Percentage contribution of different close contacts to the Hirshfeld surface of forms (I), (I·0.5MeOH), I in (I·0.5MeOH) and MeOH in (I·0.5MeOH).

The complete two-dimensional fingerprint plots for (I), (I·0.5MeOH) and, for additional comparison, I in (I·0.5MeOH), along with the decomposed two-dimensional plots for the indicated inter­actions are presented in Fig. 6 ▸, while the percentage contributions are represented graphically in Fig. 7 ▸. As mentioned previously, mol­ecules of (I) in its unsolvated and solvated forms are governed by similar inter­molecular close contacts which mainly comprise non-hydrogen-bond inter­actions. Specifically, H⋯H, being the most dominant inter­action among all, *ca* 57.3% in (I) and 55.4% in (I·0.5MeOH), forms a forceps-like fingerprint in (I), by contrast to the distinctive spike of (I·0.5MeOH), Fig. 6 ▸ *b*. It is noted there is not much to distinguish the fingerprint patterns due to C⋯H/H⋯C, Fig. 6 ▸ *c*. This observation is vindicated by the near equivalence of the sums of the *d*_e_ + *d*_i_ distances of ∼2.70 Å for (I) and ∼2.64 Å for (I·0.5MeOH) and with the relative contributions of approximately 23.3 and 23.8% to the overall surface areas, respectively. However, a marked difference is observed in the corresponding pincers-like fingerprint plots due to S⋯H/H⋯S interactions, Fig. 6 ▸ *d*. Thus, the plot for (I) displays a sum of inter­molecular contact distance *d*_e_ + *d*_i_ of ∼2.88 Å, originating from weak phenyl-C–H⋯S contacts. For the solvate, a mixed inter­action mode is evident from the asymmetric fingerprint plot indicating inter­actions between two chemically and crystallographically distinct mol­ecules, *i.e*. the relatively strong solvent⋯solute methyl-C—H⋯S inter­action with the sum of *d*_e_ + *d*_i_ distances being ∼2.42 Å coupled with a weak meth­oxy-C—H⋯S contact with *d*_e_ + *d*_i_ = ∼3.1 Å. Such inter­actions contribute roughly 3.2% (S⋯H–solvent) and 1.1% (S⋯H–meth­oxy) to the total 4.3% to the overall Hirshfeld surface of I in (I·0.5MeOH) compared to a ∼7.5% contribution in (I). Mol­ecule (I) does not forms any meaningful contacts through O⋯H/H⋯O owing to their long contact distances despite these contacts constituting approximately 2.4% of the overall contacts on the Hirshfeld surface, Fig. 6 ▸ *e*. Upon crystallization with methanol solvent, the overall contribution increases to 6.4% with the sum of *d*_e_ + *d*_i_ of ∼2.50 Å which is considered longer than typical O⋯H inter­actions with distances of ∼2.14 Å (Gavezzotti, 2016 ▸).

## Database survey   

As mentioned in the *Chemical context*, there are over 70 mol­ecular structures in the crystallographic literature (Groom *et al.*, 2016 ▸) based on the general formula *R*_3_PAu[SC(O*R*′)=N*R*′′ for *R*, *R*′ and *R*′′ = alkyl, aryl. The present structural pair, (I) and (I·0.5MeOH) represents the second example of solvatomorphism, with the prototype compound Ph_3_PAu[SC(OMe)=NPh (Hall *et al.*, 1993 ▸) being also found in a chloro­form solvate (Kuan *et al.*, 2008 ▸). The common feature of all four mol­ecules is the presence of intra­molecular Au⋯O inter­actions. Very recently, a polymorph of Ph_3_PAu[SC(OEt)=NPh has been reported (Yeo *et al.*, 2016 ▸) in which there has been a dramatic conformational change compared with the previously described form (Hall & Tiekink, 1993 ▸). While the latter features the normally observed Au⋯O inter­action, the new form features intra­molecular Au⋯π (Caracelli *et al.*, 2013 ▸) inter­actions. It was suggested that the crystallization conditions determined the conformation with that featuring the Au⋯π inter­actions being the thermodynamic outcome (Yeo *et al.*, 2015 ▸, 2016 ▸).

## Synthesis and crystallization   

IR spectra were obtained on a Perkin–Elmer Spectrum 400 FT Mid-IR/Far-IR spectrophotometer from 4000 to 400 cm^−1^; abbreviation: *s*, strong. The ^1^H NMR spectrum was recorded in CDCl_3_ on a Bruker Avance 400 MHz NMR spectrometer with chemical shifts relative to tetra­methyl­silane; abbreviations for NMR assignments: *s*, singlet; *d*, doublet; *t*, triplet; *m*, multiplet.

**Preparation of (I):** NaOH (Merck; 0.20 mmol, 0.008 g) in MeOH (Merck; 1 ml) was added to a suspension of Ph_3_PAuCl (0.20 mmol, 0.100 g) in MeOH (Merck; 10 ml), followed by addition of the thio­carbamide, MeOC(=S)N(H)(*o*-tol) (0.20 mmol, 0.036 g), prepared following literature precedents (Ho *et al.*, 2005 ▸), in MeOH (10 ml). The resulting mixture was stirred for 2 h at 323 K. The solution was left for slow evaporation at room temperature, yielding colourless blocks after 2 weeks. Yield: 0.109 g (85%). M.p. 389–391 K.

IR (cm^−1^): 1435 (*s*) (C=N), 1132 (*s*) (C—O), 1100 (*s*) (C—S). ^1^H NMR (400 MHz, CDCl_3_, 298 K): δ 7.53–7.39 (*m*, *br*, 15H, Ph_3_P), 6.86 (*d*, 1H, *o*-tol-H4, *J* = 6.24 Hz), 6.85 (*t*, 1H, *o*-tol-H3, *J* = 6.16 Hz), 6.73 (*d*, 1H, *o*-tol-H1, *J* = 7.70 Hz), 6.54 (*t*, 1H, *o*-tol-H2, *J* = 7.16 Hz), 3.93 (*s*, 3H, OMe), 2.11 (*s*, 3H, *o*-tol-Me) p.p.m.

## Refinement   

Crystal data, data collection and structure refinement details are summarized in Table 4 ▸. The carbon-bound H atoms were placed in calculated positions (C—H = 0.95–0.98 Å) and were included in the refinement in the riding-model approximation, with *U*_iso_(H) set to 1.2–1.5*U*_equiv_(C). Owing to poor agreement, a number of reflections, *i.e*. (0 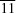 4), (9 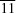 5), (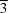 3 12), (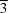 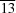 7), (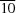 11 2), (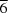 10 1), (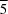 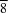 4), (7 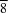 6), (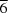 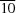 9), (4 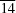 2), (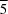 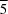 14), (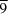 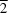 15), (6 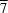 7) and (5 8 5), were omitted from the final cycles of refinement. The maximum and minimum residual electron density peaks of 0.97 and 1.14 e Å^−3^, respectively, were located 0.80 and 0.85 Å from the Au atom.

## Supplementary Material

Crystal structure: contains datablock(s) I, global. DOI: 10.1107/S2056989016014419/hb7616sup1.cif

Structure factors: contains datablock(s) I. DOI: 10.1107/S2056989016014419/hb7616Isup2.hkl

CCDC reference: 1503822

Additional supporting information: crystallographic information; 3D view; checkCIF report

## Figures and Tables

**Table 1:** Selected geometric data (Å, °) for (I) and (I·0.5MeOH)^*a*^

Parameter	(I)	(I·0.5MeOH)
Au—S1	2.3114 (6)	2.3009 (17)
Au—P1	2.2529 (6)	2.2558 (15)
C1—S1	1.768 (3)	1.751 (7)
C1—O1	1.359 (3)	1.356 (9)
C1—N1	1.260 (3)	1.260 (8)
Au⋯O1	3.040 (2)	3.093 (5)
S1—Au—P1	177.61 (2)	175.52 (6)
Au—S1—C1	103.14 (9)	105.0 (2)
C1—O1—C9	114.9 (2)	116.3 (5)
C1—N1—C2	121.4 (2)	121.2 (6)
S1—C1—O1	113.38 (18)	113.5 (4)
S1—C1—N1	125.9 (2)	126.0 (6)
O1—C1—N1	120.7 (2)	120.5 (6)

Note: (*a*) Kuan *et al.* (2008 ▸).

**Table 2:** Hydrogen-bond geometry (Å, °) *Cg*1 and *Cg*2 are the centroids of the (C2–C7) and (C22–C27) rings, respectively.

*D*—H⋯*A*	*D*—H	H⋯*A*	*D*⋯*A*	*D*—H⋯*A*
C8—H8*C*⋯*Cg*1^i^	0.98	2.73	3.481 (3)	134
*Cg*2⋯*Cg*2^ii^	–	–	3.8033 (17)	–

Symmetry codes: (i) 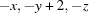; (ii) 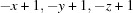.

**Table 3:** Physiochemical properties for (I), (I·0.5MeOH), and I and MeOH in (I·0.5MeOH)

Parameter	(I)	(I·0.5MeOH)	
		I	MeOH
Volume, *V* (Å^3^)	590.16	637.63	591.04
Surface area, *A* (Å^2^)	514.76	543.39	512.10
*A*:*V* (Å^−1^)	0.87	0.85	0.87
Globularity, *G*	0.661	0.659	0.665
Asphericity, Ω	0.159	0.100	0.138
Density (g cm^−1^)	1.767	1.658	–
Packing index (%)	68.2	67.3	–

**Table 4:** Experimental details

Crystal data	
Chemical formula	[Au(C_9_H_10_NOS)(C_18_H_15_P)]
*M*_r_	639.47
Crystal system, space group	Triclinic, *P* 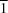
Temperature (K)	100
*a*, *b*, *c* (Å)	9.3884 (8), 10.0610 (8), 13.3572 (11)
α, β, γ (°)	96.194 (1), 102.487 (1), 99.443 (1)
*V* (Å^3^)	1201.60 (17)
*Z*	2
Radiation type	Mo *K*α
μ (mm^−1^)	6.30
Crystal size (mm)	0.30 × 0.11 × 0.09

Data collection	
Diffractometer	Bruker *SMART* *APEX* CCD
Absorption correction	Multi-scan (*SADABS*; Sheldrick, 1996 ▸)
*T*_min_, *T*_max_	0.368, 0.746
No. of measured, independent and observed [*I* > 2σ(*I*)] reflections	18394, 7189, 6714
*R*_int_	0.031
(sin θ/λ)_max_ (Å^−1^)	0.716

Refinement	
*R*[*F* ^2^ > 2σ(*F* ^2^)], *wR*(*F* ^2^), *S*	0.023, 0.055, 1.04
No. of reflections	7189
No. of parameters	291
H-atom treatment	H-atom parameters constrained
Δρ_max_, Δρ_min_ (e Å^−3^)	0.97, −1.14

Computer programs: *SMART* and *SAINT* (Bruker, 2007 ▸), *SHELXS97* (Sheldrick, 2008 ▸), *SHELXL2014/7* (Sheldrick, 2015 ▸), *ORTEP-3 for Windows* (Farrugia, 2012 ▸), *QMol* (Gans & Shalloway, 2001 ▸), *DIAMOND* (Brandenburg, 2006 ▸) and *publCIF* (Westrip, 2010 ▸).

## supplementary crystallographic information

### Crystal data

**Table:**

[Au(C_9_H_10_NOS)(C_18_H_15_P)]	*Z* = 2
*M**_r_* = 639.47	*F*(000) = 624
Triclinic, *P*1	*D*_x_ = 1.767 Mg m^−^^3^
*a* = 9.3884 (8) Å	Mo *K*α radiation, λ = 0.71073 Å
*b* = 10.0610 (8) Å	Cell parameters from 9945 reflections
*c* = 13.3572 (11) Å	θ = 2.3–30.6°
α = 96.194 (1)°	µ = 6.30 mm^−^^1^
β = 102.487 (1)°	*T* = 100 K
γ = 99.443 (1)°	Block, colourless
*V* = 1201.60 (17) Å^3^	0.30 × 0.11 × 0.09 mm

### Data collection

**Table:**

Bruker SMART APEX CCD diffractometer	7189 independent reflections
Radiation source: fine-focus sealed tube	6714 reflections with *I* > 2σ(*I*)
Graphite monochromator	*R*_int_ = 0.031
φ and ω scans	θ_max_ = 30.6°, θ_min_ = 1.6°
Absorption correction: multi-scan (SADABS; Sheldrick, 1996)	*h* = −13→13
*T*_min_ = 0.368, *T*_max_ = 0.746	*k* = −14→14
18394 measured reflections	*l* = −18→19

### Refinement

**Table:**

Refinement on *F*^2^	0 restraints
Least-squares matrix: full	Hydrogen site location: inferred from neighbouring sites
*R*[*F*^2^ > 2σ(*F*^2^)] = 0.023	H-atom parameters constrained
*wR*(*F*^2^) = 0.055	*w* = 1/[σ^2^(*F*_o_^2^) + (0.0186*P*)^2^ + 1.2573*P*] where *P* = (*F*_o_^2^ + 2*F*_c_^2^)/3
*S* = 1.04	(Δ/σ)_max_ = 0.002
7189 reflections	Δρ_max_ = 0.97 e Å^−^^3^
291 parameters	Δρ_min_ = −1.14 e Å^−^^3^

### Special details

**Table:**

Geometry. All esds (except the esd in the dihedral angle between two l.s. planes) are estimated using the full covariance matrix. The cell esds are taken into account individually in the estimation of esds in distances, angles and torsion angles; correlations between esds in cell parameters are only used when they are defined by crystal symmetry. An approximate (isotropic) treatment of cell esds is used for estimating esds involving l.s. planes.

### Fractional atomic coordinates and isotropic or equivalent isotropic displacement parameters (Å^2^)

**Table:**

	*x*	*y*	*z*	*U*_iso_*/*U*_eq_	
Au	0.62037 (2)	0.84221 (2)	0.31176 (2)	0.01464 (3)	
S1	0.47523 (7)	0.98235 (6)	0.23056 (5)	0.01606 (12)	
P1	0.76792 (7)	0.70692 (7)	0.38621 (5)	0.01288 (12)	
O1	0.3113 (2)	0.73724 (19)	0.17215 (15)	0.0192 (4)	
N1	0.2275 (3)	0.8941 (2)	0.07556 (17)	0.0173 (4)	
C1	0.3230 (3)	0.8657 (3)	0.1480 (2)	0.0155 (5)	
C2	0.2349 (3)	1.0288 (3)	0.05179 (19)	0.0142 (5)	
C3	0.1468 (3)	1.1117 (3)	0.09059 (19)	0.0146 (5)	
C4	0.1448 (3)	1.2394 (3)	0.0581 (2)	0.0171 (5)	
H4	0.0852	1.2966	0.0834	0.020*	
C5	0.2279 (3)	1.2843 (3)	−0.0102 (2)	0.0189 (5)	
H5	0.2256	1.3714	−0.0312	0.023*	
C6	0.3149 (3)	1.2004 (3)	−0.0478 (2)	0.0185 (5)	
H6	0.3728	1.2305	−0.0941	0.022*	
C7	0.3170 (3)	1.0733 (3)	−0.0176 (2)	0.0171 (5)	
H7	0.3751	1.0159	−0.0444	0.021*	
C8	0.0546 (3)	1.0623 (3)	0.1631 (2)	0.0197 (5)	
H8A	−0.0207	1.1186	0.1661	0.030*	
H8B	0.1189	1.0691	0.2325	0.030*	
H8C	0.0055	0.9672	0.1379	0.030*	
C9	0.1757 (3)	0.6439 (3)	0.1169 (2)	0.0254 (6)	
H9A	0.1726	0.5565	0.1432	0.038*	
H9B	0.1731	0.6299	0.0427	0.038*	
H9C	0.0897	0.6822	0.1275	0.038*	
C10	0.7737 (3)	0.5607 (3)	0.29563 (19)	0.0141 (4)	
C11	0.8969 (3)	0.4975 (3)	0.3086 (2)	0.0188 (5)	
H11	0.9803	0.5322	0.3650	0.023*	
C12	0.8984 (4)	0.3844 (3)	0.2396 (2)	0.0227 (6)	
H12	0.9836	0.3435	0.2477	0.027*	
C13	0.7749 (4)	0.3316 (3)	0.1588 (2)	0.0248 (6)	
H13	0.7743	0.2522	0.1131	0.030*	
C14	0.6526 (4)	0.3940 (3)	0.1447 (2)	0.0274 (6)	
H14	0.5689	0.3581	0.0887	0.033*	
C15	0.6521 (3)	0.5091 (3)	0.2123 (2)	0.0202 (5)	
H15	0.5687	0.5527	0.2016	0.024*	
C16	0.9625 (3)	0.7857 (2)	0.4343 (2)	0.0157 (5)	
C17	1.0410 (3)	0.7831 (3)	0.5353 (2)	0.0185 (5)	
H17	0.9900	0.7481	0.5839	0.022*	
C18	1.1938 (3)	0.8315 (3)	0.5651 (2)	0.0211 (5)	
H18	1.2467	0.8307	0.6342	0.025*	
C19	1.2689 (3)	0.8808 (3)	0.4942 (2)	0.0217 (5)	
H19	1.3737	0.9109	0.5140	0.026*	
C20	1.1911 (3)	0.8863 (3)	0.3940 (2)	0.0232 (6)	
H20	1.2426	0.9221	0.3459	0.028*	
C21	1.0389 (3)	0.8399 (3)	0.3643 (2)	0.0200 (5)	
H21	0.9860	0.8447	0.2961	0.024*	
C22	0.7133 (3)	0.6418 (3)	0.49658 (19)	0.0146 (5)	
C23	0.6720 (3)	0.7323 (3)	0.5671 (2)	0.0180 (5)	
H23	0.6636	0.8215	0.5527	0.022*	
C24	0.6434 (3)	0.6919 (3)	0.6579 (2)	0.0203 (5)	
H24	0.6175	0.7540	0.7066	0.024*	
C25	0.6526 (3)	0.5605 (3)	0.6777 (2)	0.0218 (5)	
H25	0.6333	0.5331	0.7401	0.026*	
C26	0.6895 (3)	0.4696 (3)	0.6072 (2)	0.0211 (5)	
H26	0.6939	0.3795	0.6209	0.025*	
C27	0.7205 (3)	0.5091 (3)	0.5160 (2)	0.0166 (5)	
H27	0.7461	0.4465	0.4676	0.020*	

### Atomic displacement parameters (Å^2^)

**Table:**

	*U*^11^	*U*^22^	*U*^33^	*U*^12^	*U*^13^	*U*^23^
Au	0.01482 (5)	0.01725 (5)	0.01246 (5)	0.00688 (3)	0.00122 (3)	0.00319 (3)
S1	0.0148 (3)	0.0158 (3)	0.0163 (3)	0.0049 (2)	−0.0005 (2)	0.0021 (2)
P1	0.0130 (3)	0.0143 (3)	0.0110 (3)	0.0041 (2)	0.0010 (2)	0.0018 (2)
O1	0.0209 (10)	0.0144 (9)	0.0202 (9)	0.0052 (7)	−0.0014 (8)	0.0038 (7)
N1	0.0172 (11)	0.0163 (10)	0.0164 (10)	0.0045 (8)	−0.0009 (8)	0.0019 (8)
C1	0.0175 (12)	0.0149 (11)	0.0136 (11)	0.0054 (9)	0.0019 (9)	−0.0005 (9)
C2	0.0130 (11)	0.0151 (11)	0.0114 (10)	0.0026 (9)	−0.0029 (9)	0.0008 (9)
C3	0.0145 (11)	0.0173 (11)	0.0096 (10)	0.0018 (9)	−0.0002 (9)	−0.0004 (9)
C4	0.0205 (13)	0.0151 (11)	0.0153 (11)	0.0051 (10)	0.0027 (10)	0.0017 (9)
C5	0.0212 (13)	0.0167 (12)	0.0174 (12)	0.0006 (10)	0.0027 (10)	0.0046 (10)
C6	0.0173 (12)	0.0225 (13)	0.0159 (12)	0.0019 (10)	0.0051 (10)	0.0038 (10)
C7	0.0135 (11)	0.0202 (12)	0.0166 (12)	0.0048 (10)	0.0011 (9)	0.0006 (10)
C8	0.0215 (13)	0.0206 (12)	0.0159 (12)	0.0016 (10)	0.0040 (10)	0.0021 (10)
C9	0.0283 (15)	0.0145 (12)	0.0278 (15)	−0.0001 (11)	−0.0035 (12)	0.0067 (11)
C10	0.0150 (11)	0.0165 (11)	0.0110 (10)	0.0025 (9)	0.0036 (9)	0.0021 (9)
C11	0.0203 (13)	0.0209 (12)	0.0153 (12)	0.0076 (10)	0.0029 (10)	0.0005 (10)
C12	0.0295 (15)	0.0221 (13)	0.0215 (13)	0.0125 (12)	0.0106 (12)	0.0045 (11)
C13	0.0360 (17)	0.0183 (13)	0.0184 (13)	0.0025 (12)	0.0077 (12)	−0.0037 (10)
C14	0.0290 (16)	0.0279 (15)	0.0175 (13)	−0.0040 (12)	0.0000 (12)	−0.0049 (11)
C15	0.0139 (12)	0.0267 (14)	0.0183 (12)	0.0014 (10)	0.0026 (10)	0.0026 (10)
C16	0.0189 (12)	0.0112 (10)	0.0151 (11)	0.0038 (9)	0.0003 (10)	−0.0004 (9)
C17	0.0174 (12)	0.0191 (12)	0.0173 (12)	0.0018 (10)	0.0020 (10)	0.0027 (10)
C18	0.0157 (12)	0.0208 (13)	0.0214 (13)	−0.0006 (10)	−0.0036 (10)	0.0022 (10)
C19	0.0147 (12)	0.0192 (12)	0.0276 (14)	−0.0016 (10)	0.0039 (11)	−0.0027 (11)
C20	0.0238 (14)	0.0206 (13)	0.0256 (14)	−0.0014 (11)	0.0123 (12)	0.0016 (11)
C21	0.0216 (13)	0.0194 (12)	0.0183 (12)	0.0037 (10)	0.0030 (10)	0.0038 (10)
C22	0.0140 (11)	0.0157 (11)	0.0135 (11)	0.0029 (9)	0.0021 (9)	0.0014 (9)
C23	0.0178 (12)	0.0174 (12)	0.0174 (12)	0.0031 (10)	0.0026 (10)	−0.0005 (9)
C24	0.0187 (13)	0.0247 (13)	0.0160 (12)	0.0032 (11)	0.0041 (10)	−0.0014 (10)
C25	0.0176 (13)	0.0326 (15)	0.0160 (12)	0.0038 (11)	0.0051 (10)	0.0062 (11)
C26	0.0217 (13)	0.0199 (13)	0.0237 (14)	0.0038 (10)	0.0077 (11)	0.0066 (10)
C27	0.0167 (12)	0.0162 (11)	0.0166 (12)	0.0051 (9)	0.0025 (10)	0.0014 (9)

### Geometric parameters (Å, º)

**Table:**

Au—P1	2.2529 (6)	C11—H11	0.9500
Au—S1	2.3114 (6)	C12—C13	1.387 (4)
S1—C1	1.768 (3)	C12—H12	0.9500
P1—C16	1.812 (3)	C13—C14	1.384 (5)
P1—C22	1.814 (3)	C13—H13	0.9500
P1—C10	1.817 (3)	C14—C15	1.391 (4)
O1—C1	1.359 (3)	C14—H14	0.9500
O1—C9	1.449 (3)	C15—H15	0.9500
N1—C1	1.260 (3)	C16—C17	1.396 (4)
N1—C2	1.419 (3)	C16—C21	1.399 (4)
C2—C7	1.392 (4)	C17—C18	1.391 (4)
C2—C3	1.403 (4)	C17—H17	0.9500
C3—C4	1.402 (4)	C18—C19	1.383 (4)
C3—C8	1.503 (4)	C18—H18	0.9500
C4—C5	1.388 (4)	C19—C20	1.391 (4)
C4—H4	0.9500	C19—H19	0.9500
C5—C6	1.394 (4)	C20—C21	1.384 (4)
C5—H5	0.9500	C20—H20	0.9500
C6—C7	1.383 (4)	C21—H21	0.9500
C6—H6	0.9500	C22—C27	1.396 (4)
C7—H7	0.9500	C22—C23	1.398 (4)
C8—H8A	0.9800	C23—C24	1.386 (4)
C8—H8B	0.9800	C23—H23	0.9500
C8—H8C	0.9800	C24—C25	1.388 (4)
C9—H9A	0.9800	C24—H24	0.9500
C9—H9B	0.9800	C25—C26	1.379 (4)
C9—H9C	0.9800	C25—H25	0.9500
C10—C11	1.396 (4)	C26—C27	1.396 (4)
C10—C15	1.394 (4)	C26—H26	0.9500
C11—C12	1.389 (4)	C27—H27	0.9500
			
P1—Au—S1	177.61 (2)	C13—C12—C11	119.7 (3)
C1—S1—Au	103.14 (9)	C13—C12—H12	120.2
C16—P1—C22	104.74 (12)	C11—C12—H12	120.2
C16—P1—C10	102.91 (12)	C14—C13—C12	120.2 (3)
C22—P1—C10	107.07 (12)	C14—C13—H13	119.9
C16—P1—Au	115.26 (8)	C12—C13—H13	119.9
C22—P1—Au	113.63 (9)	C13—C14—C15	120.2 (3)
C10—P1—Au	112.28 (9)	C13—C14—H14	119.9
C1—O1—C9	114.9 (2)	C15—C14—H14	119.9
C1—N1—C2	121.4 (2)	C14—C15—C10	120.1 (3)
N1—C1—O1	120.7 (2)	C14—C15—H15	120.0
N1—C1—S1	125.9 (2)	C10—C15—H15	120.0
O1—C1—S1	113.38 (18)	C17—C16—C21	119.2 (3)
C7—C2—C3	120.2 (2)	C17—C16—P1	122.4 (2)
C7—C2—N1	120.3 (2)	C21—C16—P1	118.1 (2)
C3—C2—N1	119.2 (2)	C18—C17—C16	120.2 (3)
C4—C3—C2	118.3 (2)	C18—C17—H17	119.9
C4—C3—C8	121.4 (2)	C16—C17—H17	119.9
C2—C3—C8	120.4 (2)	C19—C18—C17	120.1 (3)
C5—C4—C3	121.5 (2)	C19—C18—H18	120.0
C5—C4—H4	119.3	C17—C18—H18	120.0
C3—C4—H4	119.3	C18—C19—C20	120.1 (3)
C4—C5—C6	119.3 (2)	C18—C19—H19	119.9
C4—C5—H5	120.3	C20—C19—H19	119.9
C6—C5—H5	120.3	C21—C20—C19	120.0 (3)
C7—C6—C5	120.1 (2)	C21—C20—H20	120.0
C7—C6—H6	120.0	C19—C20—H20	120.0
C5—C6—H6	120.0	C20—C21—C16	120.4 (3)
C6—C7—C2	120.6 (2)	C20—C21—H21	119.8
C6—C7—H7	119.7	C16—C21—H21	119.8
C2—C7—H7	119.7	C27—C22—C23	119.9 (2)
C3—C8—H8A	109.5	C27—C22—P1	122.34 (19)
C3—C8—H8B	109.5	C23—C22—P1	117.6 (2)
H8A—C8—H8B	109.5	C24—C23—C22	120.0 (3)
C3—C8—H8C	109.5	C24—C23—H23	120.0
H8A—C8—H8C	109.5	C22—C23—H23	120.0
H8B—C8—H8C	109.5	C25—C24—C23	119.9 (3)
O1—C9—H9A	109.5	C25—C24—H24	120.0
O1—C9—H9B	109.5	C23—C24—H24	120.0
H9A—C9—H9B	109.5	C26—C25—C24	120.4 (3)
O1—C9—H9C	109.5	C26—C25—H25	119.8
H9A—C9—H9C	109.5	C24—C25—H25	119.8
H9B—C9—H9C	109.5	C25—C26—C27	120.5 (3)
C11—C10—C15	119.2 (2)	C25—C26—H26	119.8
C11—C10—P1	121.3 (2)	C27—C26—H26	119.8
C15—C10—P1	119.5 (2)	C22—C27—C26	119.3 (2)
C12—C11—C10	120.6 (3)	C22—C27—H27	120.4
C12—C11—H11	119.7	C26—C27—H27	120.4
C10—C11—H11	119.7		
			
C2—N1—C1—O1	177.6 (2)	C11—C10—C15—C14	1.7 (4)
C2—N1—C1—S1	0.8 (4)	P1—C10—C15—C14	−177.5 (2)
C9—O1—C1—N1	−6.4 (4)	C22—P1—C16—C17	2.7 (2)
C9—O1—C1—S1	170.9 (2)	C10—P1—C16—C17	−109.1 (2)
Au—S1—C1—N1	−167.2 (2)	Au—P1—C16—C17	128.3 (2)
Au—S1—C1—O1	15.8 (2)	C22—P1—C16—C21	176.7 (2)
C1—N1—C2—C7	88.2 (3)	C10—P1—C16—C21	64.9 (2)
C1—N1—C2—C3	−98.3 (3)	Au—P1—C16—C21	−57.6 (2)
C7—C2—C3—C4	−0.4 (4)	C21—C16—C17—C18	−1.2 (4)
N1—C2—C3—C4	−173.9 (2)	P1—C16—C17—C18	172.8 (2)
C7—C2—C3—C8	178.3 (2)	C16—C17—C18—C19	−0.9 (4)
N1—C2—C3—C8	4.7 (4)	C17—C18—C19—C20	2.2 (4)
C2—C3—C4—C5	−0.3 (4)	C18—C19—C20—C21	−1.4 (4)
C8—C3—C4—C5	−178.9 (2)	C19—C20—C21—C16	−0.7 (4)
C3—C4—C5—C6	0.3 (4)	C17—C16—C21—C20	2.0 (4)
C4—C5—C6—C7	0.5 (4)	P1—C16—C21—C20	−172.3 (2)
C5—C6—C7—C2	−1.2 (4)	C16—P1—C22—C27	−92.1 (2)
C3—C2—C7—C6	1.1 (4)	C10—P1—C22—C27	16.8 (3)
N1—C2—C7—C6	174.6 (2)	Au—P1—C22—C27	141.3 (2)
C16—P1—C10—C11	28.4 (2)	C16—P1—C22—C23	83.8 (2)
C22—P1—C10—C11	−81.7 (2)	C10—P1—C22—C23	−167.4 (2)
Au—P1—C10—C11	152.94 (19)	Au—P1—C22—C23	−42.8 (2)
C16—P1—C10—C15	−152.5 (2)	C27—C22—C23—C24	2.3 (4)
C22—P1—C10—C15	97.5 (2)	P1—C22—C23—C24	−173.7 (2)
Au—P1—C10—C15	−27.9 (2)	C22—C23—C24—C25	−1.4 (4)
C15—C10—C11—C12	−0.2 (4)	C23—C24—C25—C26	−0.2 (4)
P1—C10—C11—C12	178.9 (2)	C24—C25—C26—C27	1.0 (4)
C10—C11—C12—C13	−1.8 (4)	C23—C22—C27—C26	−1.5 (4)
C11—C12—C13—C14	2.3 (5)	P1—C22—C27—C26	174.3 (2)
C12—C13—C14—C15	−0.8 (5)	C25—C26—C27—C22	−0.1 (4)
C13—C14—C15—C10	−1.2 (4)		

### Hydrogen-bond geometry (Å, º)

Cg1 and Cg2 are the centroids of the (C2–C7) and (C22–C27) rings, respectively.

**Table:**

*D*—H···*A*	*D*—H	H···*A*	*D*···*A*	*D*—H···*A*
C8—H8*C*···*Cg*1^i^	0.98	2.73	3.481 (3)	134
*Cg*2—–···*Cg*2^ii^	–	–	3.8033 (17)	–

Symmetry codes: (i) −*x*, −*y*+2, −*z*; (ii) −*x*+1, −*y*+1, −*z*+1.
